# Multimodality imaging in a late septic infection of aortic graft

**DOI:** 10.1259/bjrcr.20150396

**Published:** 2016-05-12

**Authors:** Monika Arzanauskaite, Antanas Jankauskas, Reda Arzanauskiene, Evaldas Keleras

**Affiliations:** ^1^ Radiology Department, Hospital of Lithuanian University of Health Sciences, Kaunas, Lithuania; ^2^ Cardiology Department, Hospital of Lithuanian University of Health Sciences, Kaunas, Lithuania

## Abstract

A 70-year-old diabetic female patient presented with fatigue, headaches, hallucinations and shivers following a history of sinusitis and ophthalmitis. She had an aortic surgery performed 7 years ago for a stenotic and regurgitant aortic valve with aneurysm of the ascending aorta. Work-up brain MRI revealed septic–embolic encephalitis. Multimodality cardiovascular imaging showed abnormal anterior wall of the ascending aortic graft with vegetation extending into the lumen. Blood culture was only positive for *Aggregatibacter actinomycetemcomitans,* an uncommon cause of infective endocarditis. During aortic surgery, the intraluminal vegetation with suppurated perigraft tissue was confirmed.

## Summary

**Figure 1.**
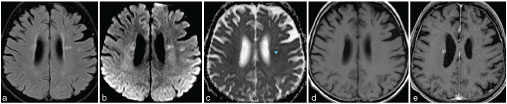
Brain MRI. (a) Axial slice of *T*_2_ weighted dark fluid sequence with fat suppression. One of the multiple small hyperintense lesions is shown in the periventricular area. (b) Axial slice of high-b-value diffusion sequence. The hyperintense signal of the periventricular lesion is noteworthy. (c) Axial image of apparent diffusion coefficient map. The signal intensity of the same periventricular lesion (arrowhead) is low, therefore, consistent with diffusion restriction. ) Axial slices of *T*_1_ weighted sequence (d) before and (e) after contrast administration. The enhancement of the small periventricular lesion depicted in the previous images is noteworthy—the absence of ring pattern indicates that there is no fully formed abscess yet.

We report a case of a 70-year-old diabetic female patient presenting with fatigue, headaches, hallucinations, shivers and history of arterial hypertension and myocardial infarction. She had the Bentall procedure performed 7 years ago for a severely stenotic and moderately regurgitant aortic valve with aneurysm of the ascending aorta. The patient had a tooth extracted months before the current presentation, which was complicated by sinusitis that was treated successfully. This was followed by acute ophthalmitis 5 months later. On current presentation, she was hospitalized to exclude encephalitis. An MRI of the brain revealed multiple small brain parenchymal lesions distributed in the anterior and middle circulation territories, consistent with septic–embolic encephalitis. See Figures 1a-e that show a round, enhancing, focal periventricular lesion (Figure 1e) with diffusion restriction (Figure 1b,c).

**Figure 2.**
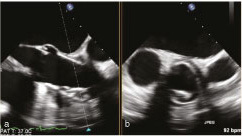
Transoesophageal echocardiography. Cross-sectional plane of the intraluminal structure in the ascending aortic graft.

**Figure 3.**
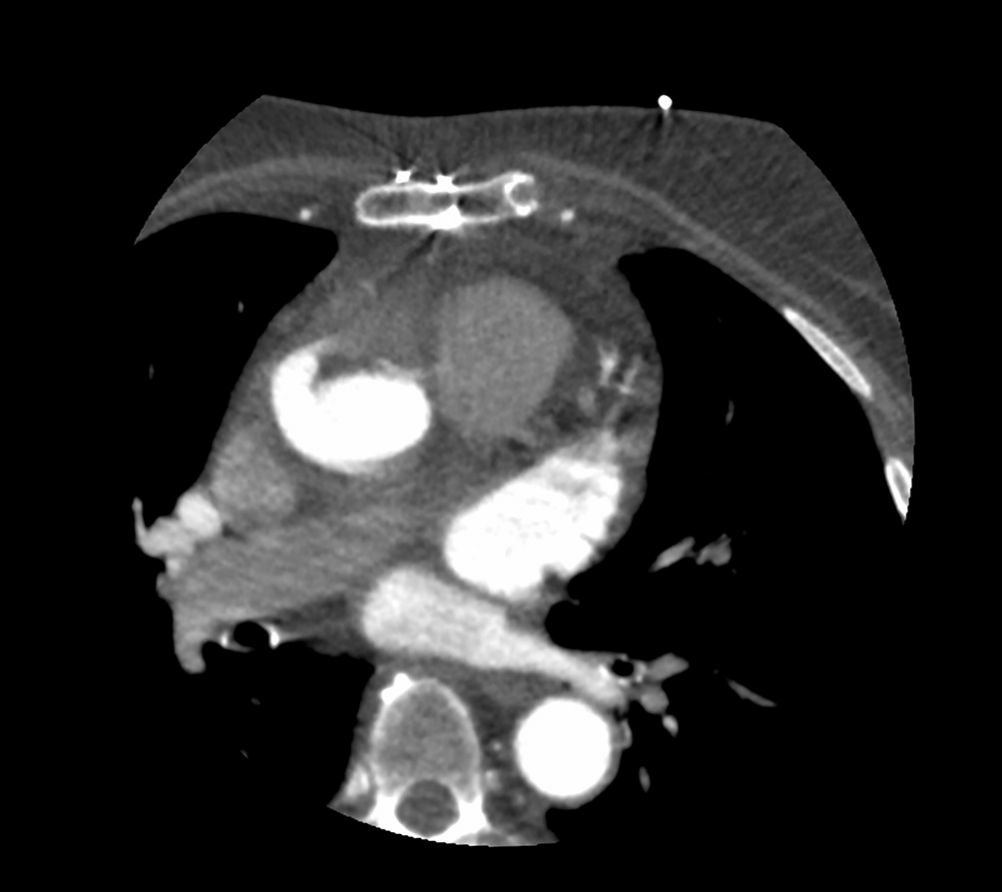
Axial slice of CT angiography. The anterior wall of the graft is irregularly thickened with a hypodense component extending into the lumen.

**Figure 4.**
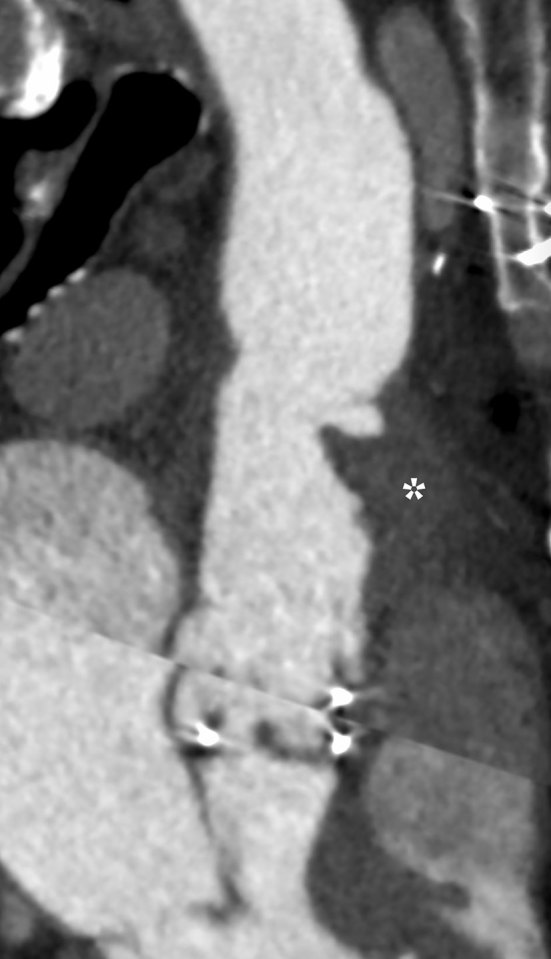
Curved multiplanar reconstruction of CT angiography. The intraluminal vegetation arises from the thickened wall (asterisk) of the graft.

On transoesophageal echocardiography, an intraluminal structure in the ascending aorta was noted (Figure 2, Supplementary Video A). For better assessment of the aortic wall, a single-beat prospective ECG-gated helical cardiac CT angiography was performed, which showed thickening and irregularity of the anterior wall of the ascending aortic graft with an intraluminal hypodense formation (Figures 3 and 4). The periaortic tissue mildly enhanced after contrast administration (only arterial contrast phase was performed). The aortic valve was not affected by the vegetation.

**Figure 5.**
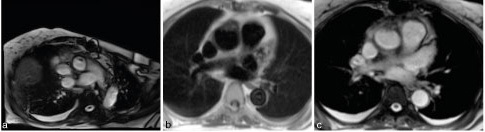
Cardiovascular MR. (a) Transaxial steady-state free precession cine of the ascending aorta shows the intraluminal mobile mass. (b) Axial slice of axial spin echo (half-Fourier acquisition single-shot turbo spin-echo) sequence at the same position as the CT image in Figure 3. The irregularly thickened anterior wall of the aortic graft with intraluminal components is noteworthy. (c) Axial slice of gradient echo (true fast imaging with steady-state free precession) sequence at the same position as the previous image confirms the irregularly thickened anterior wall of the aortic graft with vegetation extending into the lumen.

Cardiovascular MR study confirmed the ultrasound and CT findings, showing thickened anterior wall of the graft with vegetation-like luminal extension (Figure 5a, Supplementary Video B). The thickened aortic graft lesion and the intraluminal component showed heterogeneous MRI signal intensity, which may reflect an early stage of abscess formation (Figure 5b,c).

After multiple negative results, the blood culture was only positive for *Aggregatibacter actinomycetemcomitans*, a Gram-negative microorganism that belongs to the HACEK group of infective endocarditis (HACEK is an abbreviation of the initials of unusual organisms that cause infective endocarditis: *Haemophilus, Aggregatibacter, Cardiobacterium, Eikenella and Kingella* species).

An urgent surgery of the ascending aorta was performed and the imaging findings were confirmed. The perigraft tissue was abnormal with adhesions and suppurated fibrin layers; there was an intraluminal vegetation attached to the anterior wall. The aortic valve appeared unaffected. The graft was then replaced. The patient made a full recovery after post-operative antibiotic therapy.

## Discussion

A wide spectrum of complications may arise following aorta and aortic valve repair, including post-pericardiotomy syndromes, dysfunction of the prosthesis and infective endocarditis with systemic embolization.^1^ Late graft infection following aortic surgery is uncommon, accounting for 0.9–1.9% of cases.^2,3^

Although vegetation formation on aortic graft is less frequently seen compared with the valve, it has been reported.^4,5^ Therefore, infective endocarditis should always be suspected, particularly when patients present with febrile illness. *A. actinomycetemcomitans* belongs to the HACEK group of organisms that are considered to be an uncommon cause of infective endocarditis, and often a cause of blood culture-negative disease.^6^ Imaging is extremely useful in the management of these patients.

The European Society of Cardiology guidelines recommend strict follow-up surveillance after an endovascular aortic repair. When it comes to surgical repair of the aorta, the recommendations are less clear: in cases that are stable and free from complications during the first year, imaging surveillance interval may be longer than 1–2 years.^7^

Transthoracic echocardiography is cheap, widely available and often is able to assess infective complications of the aortic valve. But the technique is operator-dependent and visualization of the ascending aorta/graft may be a challenge. Therefore, in the process of diagnosis, a transoesophageal approach offers better visualization of the valves and the ascending aorta; however, the technique is semi-invasive and requires sedating the patient. Comprehensive evaluation of the entire aorta, its wall and para-aortic structures can be better achieved by a CT scan and an MRI; both of these techniques are being increasingly used in the diagnostic approach of aortic valve surgery complications.^1^ For an accurate depiction of the aortic root and ascending aorta, a multidetector CT angiography with ECG gating should be used, as motion artefacts of this particular area can significantly degrade the image quality. The role of hybrid techniques is emerging: positron emission tomography imaging alone or ideally combined with CT may be used to document ^18^F-fludeoxyglucose uptake in the inflamed aortic wall.^7,8^

The advantages of MRI are well known and include lack of ionizing radiation, high temporal resolution and periaortic tissue characterization; therefore, this modality is strongly indicated for imaging any aortic pathology.^9^ While contrast enhancement of the thickened wall shows an active process both on CT and MRI studies, *T*_2_ weighted fat-suppressed MRI sequences may show oedema of the arterial wall, which often reflects active inflammation. However, the patient’s compliance with breath-holding sequences is of key importance for appropriate assessment.

Although unlikely, thrombus is the other differential diagnosis. The presence of abnormal perigraft tissue, positive culture, clinical setting and a very unusual place for a thrombus suggest an infectious cause.

## Learning points

1. Infective endocarditis should always be considered even many years after aortic repair.
2. Imaging is an important part of the work-up in patients with suspected infective endocarditis after aortic surgery. A number of imaging techniques are available for this purpose. While each has its own advantages and limitations, cross-sectional imaging is often required for an adequate assessment. MRI and CT scan are particularly useful for evaluation of the aortic wall, graft and periaortic tissue.
3. Intraluminal vegetations have characteristics similar to thrombi. Thickening and not uncommonly enhancing wall of the vessel or graft is the key finding.

## Consent

Informed consent was obtained from the patient for publication of this case report, including accompanying images.
